# Editorial: Global excellence in dental medicine: South America

**DOI:** 10.3389/fdmed.2023.1333268

**Published:** 2023-11-28

**Authors:** Taia Maria Berto Rezende, Marcelo Henrique Napimoga

**Affiliations:** ^1^ Departamento de Odontologia, Faculdade de Ciências de Saúde, Universidade de Brasília, Brasília, Brazil; ^2^ Pós-graduação em Ciências da Saúde, Faculdade de Ciências de Saúde, Universidade de Brasília, Brasília, Brazil; ^3^ Pós-graduação em Odontologia, Faculdade de Ciências de Saúde, Universidade de Brasília, Brasília, Brazil; ^4^ Pós-graduação em Ciências Genômicas e Biotecnologia, Universidade Católica de Brasília, Brasília, Brazil; ^5^ Laboratoy of Neuroimmune Interface of Pain Research, Faculdade São Leopoldo Mandic, Instituto de Pesquisas São Leopoldo Mandic, Campinas, Brazil

**Keywords:** tooth bleaching, implant survival rate, inhalation sedation, molar-incisor hypomineralization, dental pulp, biomineralization, inflammatory mediators

**Editorial on the Research Topic** Global excellence in dental medicine: South America

## 1. Introduction

The integration of dental and oral health into medical science and public policy is essential to the concepts of prevention, early detection, and treatment of the most common diseases, as well as many rare diseases. Frontiers in Dental Medicine has organized a series of Research Topics including recent advances in Dental Medicine and Oral Health around the world (Europe, North America and South America), showcasing academic excellence and high-quality work from international professionals and recognized researchers. Therefore, this Research Topic presents research from South America, in all areas of Dental Medicine.

According to data from the Scimago platform^1^, in 2022, 18,172 scientific articles in the field of Dentistry were published by authors from around the world. Researchers from Brazil contributed the most, totaling 1,698 articles, followed by 1,682 scientific articles published by researchers from the United States. Out of these over 18,000 published scientific articles, 2,321 came from researchers in Latin America. In this scope, articles in the field of Dentistry were published by researchers from 23 Latin American countries in 2022, with Brazil accounting for 73.15% of the entire production (1,698 manuscripts)^2^.

Looking from another perspective, that of the *H*-index, among the top 50 countries, four South American countries are among those that have published in the field of Dentistry and possess higher *H*-indices. Brazil ranks 3rd with an *H*-index of 158, Chile is in 35th place with an *H*-index of 56, Colombia is 39th with an *H*-index of 48, and finally, Argentina is 44th with an *H*-index of 42. The United States leads with an *H*-index of 255, followed by the United Kingdom with an *H*-index of 182.

Approximately 10% of all published articles in Frontiers in Dental Medicine are from South American authors/ institutions. This Research Topic was developed to advance our understanding of global excellence in South America regarding different topics in dental medicine. The Research Topic is comprised of opinion, mini review, case report, and original research articles, that support advances in dental medicine related to authors based at an institution in South America.

Aiming to work constantly and continuously to make Frontiers of Dental Medicine one of the most cited journals in the dental-oral-craniofacial field, we are excited to complete another Research Topic including five peer-reviewed articles.

## 2. Research topic articles

### 2.1. Opinion

An opinion paper has been included in this Research Topic, authored by Cavalli et al., which addresses the historical context, current status, and future outlook of dental bleaching. The authors discuss advancements in chemical components and the spectrum of post-operative dentin hypersensitivity, ranging from mild to severe, drawing insights from a plethora of previously published studies, encompassing both *in vitro* experiments and clinical trials. This opinion paper is designed to enrich our understanding of the cutting-edge developments, recent breakthroughs, and prospective directions in the realm of dental bleaching. The authors also underscore the forthcoming research focus in this domain, with an emphasis on mechanistic methodologies capable of unraveling the processes governing the generation and transportation of reactive oxygen species (ROS), as well as the influence of polymeric compositions on the efficacy of whitening procedures.

### 2.2. Mini review

In this Research Topic, a concise review centered on the inflammation of dental pulp is featured. In their narrative analysis, Lorencetti-Silva et al. elucidate the inflammatory response as a protective mechanism for the dentin-pulp complex, delving into the production and impact of cytokines and various signaling molecules. Subsequently, the review delves into the commencement of tissue repair, encompassing the role of pro-inflammatory cytokines, growth factors, components of the extracellular matrix, and other biologically active substances in this restorative process. Hence, this article expounds upon the formation and biomineralization of the dentin-pulp complex and examines how pro-inflammatory events can alter this response, with a particular focus on prostaglandins and leukotrienes.

### 2.3. Case report

In this Research Topic, a case report accompanied by a corrigendum has been incorporated by Molena et al. Molar-incisor hypomineralization (MIH) is considered a worldwide clinical problem with a global prevalence of 14.2%, ranging from 0.5% to 40.2% according to different studies. Owing to deficient mineralization, patients afflicted with MIH necessitate up to tenfold more dental interventions compared to those not afflicted by this condition. The rapid progression of dental caries, the challenges inherent in devising satisfactory restorative procedures, and the complexities associated with securing cooperation from pediatric patients in a dental setting collectively present formidable hurdles in managing this condition.

This paper chronicles the clinical management of six cases, involving patients between the ages of 8 and 12, who underwent treatment under inhaled sedation with nitrous oxide and oxygen. The spectrum of clinical procedures encompassed restorative, endodontic, and surgical interventions. The utilization of inhalational sedation played a pivotal role in facilitating behavioral management and enhancing the efficacy of local anesthesia during treatment. This approach proves advantageous for the clinical care of MIH-afflicted patients.

### 2.4. Original research

The extensive global utilization of dental implants, which have demonstrated a commendable rate of success, has been documented. Complications associated with dental implants have been categorized into biological and technical issues. Among the biological complications, certain risk factors encompass smoking and systemic conditions like uncontrolled diabetes mellitus (DM) and periodontitis, all of which are classified as patient-related factors that may contribute to implant failure. From a technical perspective, postgraduate training in implant dentistry equips dental students with advanced competencies.

Considering this contemporary context, Kang et al. undertook a retrospective single-center study to evaluate the survival rate of dental implants placed by postgraduate students in implant dentistry and identify potential risk factors for implant failure. The findings revealed a notably high survival rate, with no significant influence of gender, diabetes, smoking, ongoing medication usage, the type of implant connection system, implant location, prior bone graft procedures, or the type of prosthetic provision on the risk of implant failure.

## 3. Conclusion

This Research Topic brought together researchers from Latin America in the dental field. Dental research in South America has been growing, with the publication of high-impact articles and the participation of these Americans in international research. This fact has resulted in an increase in the number of researchers from South America who are among the most productive.

We hope this Research Topic will help both, researchers and clinicians, to adopt the latest knowledge into their daily work indicating that a relevant progress has been made towards utilizing a systems-based approach to support the practice of evidence-based dentistry and to improve dental treatment outcomes.

## Footnotes

^1^ https://www.scimagojr.com/countryrank.php?category=3501&year=2022

^2^https://www.scimagojr.com/countryrank.php?year=2022&region=Latin%20America&category=3501 (Accessed November 1, 2023)

